# *In-Cell* Biochemistry Using NMR Spectroscopy

**DOI:** 10.1371/journal.pone.0002571

**Published:** 2008-07-02

**Authors:** David S. Burz, Alexander Shekhtman

**Affiliations:** Department of Chemistry, State University of New York at Albany, Albany, New York, United States of America; University of Queensland, Australia

## Abstract

Biochemistry and structural biology are undergoing a dramatic revolution. Until now, mostly *in vitro* techniques have been used to study subtle and complex biological processes under conditions usually remote from those existing in the cell. We developed a novel in-cell methodology to post-translationally modify interactor proteins and identify the amino acids that comprise the interaction surface of a target protein when bound to the post-translationally modified interactors. Modifying the interactor proteins causes structural changes that manifest themselves on the interacting surface of the target protein and these changes are monitored using in-cell NMR. We show how Ubiquitin interacts with phosphorylated and non-phosphorylated components of the receptor tyrosine kinase (RTK) endocytic sorting machinery: STAM2 (Signal-transducing adaptor molecule), Hrs (Hepatocyte growth factor regulated substrate) and the STAM2-Hrs heterodimer. Ubiquitin binding mediates the processivity of a large network of interactions required for proper functioning of the RTK sorting machinery. The results are consistent with a weakening of the network of interactions when the interactor proteins are phosphorylated. The methodology can be applied to any stable target molecule and may be extended to include other post-translational modifications such as ubiquitination or sumoylation, thus providing a long-awaited leap to high resolution in cell biochemistry.

## Introduction

The ultimate goal of all structural and biochemical research is to understand how macromolecular interactions give rise to and regulate biological activity within a natural environment, *i.e.* in living cells. The challenge is formidable due to the complexity of living matter and the relative scarcity of appropriate *in vivo* methods. The specific interactions between proteins that participate in signal transduction processes are further complicated by post-translational modifications (PTM) of protein structure. PTM is a mechanism for regulating cellular processes; such modifications can alter the strength or number of interactions in which these proteins engage and/or redirect them to sub-cellular compartments as required for proper functioning [1]. For example, endocytosis of receptor tyrosine kinases (RTKs) requires tyrosine phosphorylation and monoubiquitination of the receptor and downstream components to sort endocytosed cargo for subsequent degradation (down regulation) or recycling to the cell surface [2], [3], [4].

**Figure 1:**
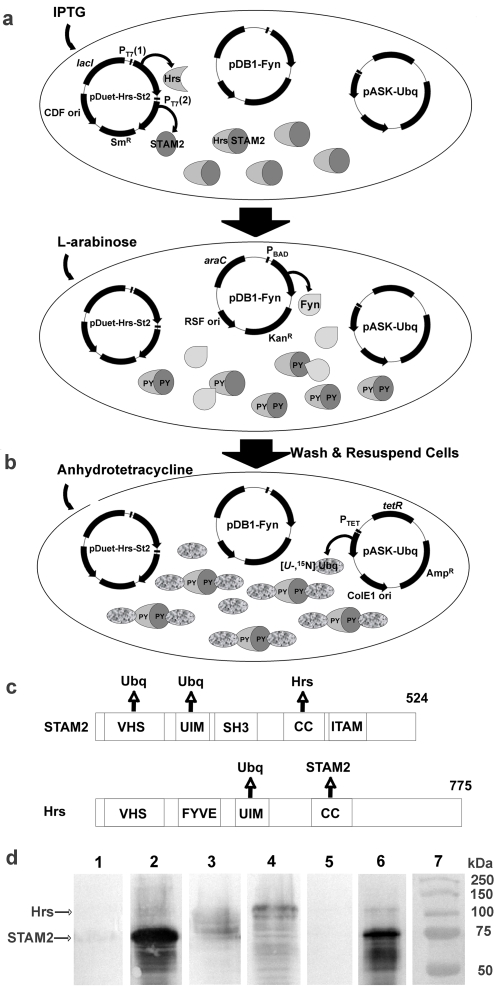
Sequential overexpression and in-cell post-translational modification of interacting proteins for STINT-NMR. *E. coli* are transformed with up to three compatible plasmids and grown overnight in LB-glucose medium containing antibiotics. The cells are washed and resuspended in label-free medium. a) IPTG is used to induce overexpression of interactor proteins, STAM2 and Hrs, which form heterodimers. L-arabinose is then used to induce overexpression of Fyn kinase, which phosphorylates tyrosine residues (PY) on the interactors. b) The cells are washed and resuspended in labeling medium. Anhydrotetracycline is used to induce overexpression of uniformly labeled [*U*-^15^N] Ubiquitin target protein, which binds to the interactors. Samples are taken as the concentration of target increases. Changes in the target protein structure are monitored using in-cell NMR spectroscopy. A sample of labeled target containing no interactor is prepared separately as a reference. *N.b.* The experiment can be performed using a single interactor protein and without post-translational modification. The protocol can also be reversed, overexpressing labeled target first, followed by interactor(s) and post-translational modification (see Materials and Methods). c) Domain structure of STAM2 and Hrs. VHS (Vps27-Hrs-Stam domain); UIM (ubiquitin interacting motif); SH3 (src homology domain 3); CC (coiled coil domain); FYVE (FYVE-finger domain); up arrows indicate Ubiquitin, STAM2 or Hrs binding domain. d) Western blot of overexpressed interactor proteins from whole cells in the absence and presence of overexpressed Fyn kinase, probed with anti-tyrosine phosphate HRP-conjugate antibody. Lane 1: STAM2; lane 2: STAM2 & Fyn kinase; lane 3: Hrs; lane 4: Hrs & Fyn kinase; lane 5: STAM2 & Hrs; lane 6: STAM2, Hrs & Fyn kinase; Lane 7: MW.

To examine the effect of PTM on protein-protein interactions that occur along this signaling pathway, we developed an in-cell biochemical methodology that, in combination with recently developed STINT-NMR (**St**ructural **Int**eractions using NMR spectroscopy) [5], allows us to directly observe the structural changes in the interaction surface of a target protein that result from phosphorylating interactor proteins. Since protein phosphorylation does not occur in *E. coli*, we can use these cells as a test tube for performing in-cell biochemistry, introducing the target protein, interactor proteins and kinase activity on separately inducible plasmids (Fig. 1ab). By employing a target protein that does not interact with endogenous bacterial proteins, the structural details of the specific interaction between these molecules can be observed in the absence and presence of post-translational modification within a cellular environment.

STINT-NMR is an in-cell technique[6], [7] for examining the structural changes in a target protein resulting from protein-protein interactions [5], [8]. Protein overexpression is first induced in uniformly labeled medium [*U*, ^15^N-] to produce a target protein containing NMR-active nuclei. The cells are washed and resuspended in non-labeling medium to induce overexpression of the interactor protein(s). We then use ^15^N-edited heteronuclear single quantum coherence (^1^H{^15^N}-HSQC) NMR experiments to monitor changes in the chemical shifts of target backbone amide nuclei as the concentration of the interactor(s) increases. In cases where the target binds to a high molecular weight interactor protein, we monitor changes in the NMR spectrum of the free target since only this species gives rise to visible peaks. Depending on the chemical exchange rate between the free and bound states of the target, affected NMR peaks, corresponding to backbone amides, can either shift, broaden their line shape or disappear completely, thereby delineating the intermolecular interaction surface between the target and the interactor(s). By changing the isotopic composition on induction, we selectively label the target while the interactor proteins remain cryptic, thus reducing NMR spectral complexity. The resulting NMR data provide a complete titration of the interaction and identify the amino acids that comprise the interaction surface of a target protein. It is important to note that in-cell titrations lack the precision of binding isotherms that are obtained *in vitro* because of the variable levels of protein expression that are inherent when using living cell, and thus, are largely qualitative. However, in each case, the same structural endpoint is attained. Tight temporal control over protein expression allows us to perform in-cell biochemistry, such as phosphorylation, by expressing a kinase domain capable of post-translationally modifying the interactor protein, off an inducible plasmid (Fig. 1ab).

To demonstrate the efficacy of this methodology, we examined changes in the binding surface of a Ubiquitin (Ubq) target that result from phosphorylating the endocytic proteins, STAM2 (Signal-transducing adaptor molecule) and Hrs (Hepatocyte growth factor-regulated tyrosine kinase substrate), both of which form homodimers with molecular weights of ∼115 kDa and ∼150 kDa, respectively. The STAM2-Hrs heterodimer (MW ∼132 kDa) directs trafficking of endocytosed, monoubiquitinated RTKs to the cell surface for recycling or to lysosomes for degradation. Several lines of evidence, mostly from the study of yeast membrane proteins, suggest that receptor sorting through endocytosis and subsequent degradation is controlled by ubiquitination of both the internalizing receptors and components of the endocytic machinery [9].

In addition to monoubiquitination, endocytic proteins undergo tyrosine phosphorylation, in some cases mediated by Src-family kinases [10], in response to cytokines or growth factor binding to RTKs. Indeed, phosphorylation of tyrosine residues on STAM2 and Hrs occurs during endocytosis. These two post-translational modifications seem to be linked since, in at least some cases, tyrosine phosphorylation is monoubiquitination-dependent [11]. The precise biological role of phosphorylation is unclear but, at least for Hrs, it was shown to be important in the cellular localization of the protein [10]. The molecular machinery that sorts endocytosed membrane proteins into the degradative pathway and away from the default recycling pathway is an area of intense research. This is because down regulation of receptor signaling provides a means to attenuate proliferation and minimize cell growth. The loss of control of down regulation can lead to rampant growth characteristic of most cancers.

STAM2 and Hrs have a modular domain architecture that allows them to participate in multiple protein-protein interactions and to bind the surrounding lipid bilayer[12] (Fig. 1c). Free Ubiquitin and monoubiquitinated proteins bind to the Ubiquitin interacting motif (UIM) present on STAM2 and Hrs [13], and to the N-terminal VHS domain of STAM2 [14]. STAM2 and Hrs are also monoubiquitinated, possibly at multiple sites, in a signal-dependent manner that requires the function of the UIM. Thus, UIM-containing monoubiquitinated protein may further assemble other monoubiquitinated and UIM-containing proteins in receptor-bound macromolecular complexes that contribute to the events leading to intracellular signal transduction and to receptor sorting in endocytic vesicles.

The main objectives of the experiments presented here are to demonstrate that we can regulate the post-translational modification of overexpressed proteins in bacterial cells, a process we dub in-cell biochemistry, and to identify structural changes in protein interaction surfaces due to presence of PTMs using in-cell NMR spectroscopy. These objectives were accomplished by successfully tyrosine phosphorylating STAM2 and Hrs (Fig. 1d), both of which undergo this post-translational modification to function in the endocytotic pathway, and by identifying changes in the interaction surfaces of Ubiquitin when bound to these post-translationally modified proteins by using STINT-NMR.

## Results

### In-cell spectrum of Ubiquitin is distinct from that obtained in vitro

There are noticeable differences between the solution and in-cell NMR spectra of free Ubiquitin, with only 86% of the Ubiquitin peaks observed in solution found within 0.1 ppm of those observed in cells [15]. To rule out the possibility that the NMR spectrum of Ubiquitin is due to extracellular protein [16], [17], after obtaining the in-cell NMR spectrum, the cells were centrifuged and the supernatant was examined. No NMR spectrum of Ubiquitin was observed above noise level (Fig. S1), implying that there is no leakage or cell lysis occurring during the time it takes to acquire the NMR spectrum.

### Binding non-phosphorylated interactor proteins creates unique contact surfaces on Ubiquitin

**Figure 2:**
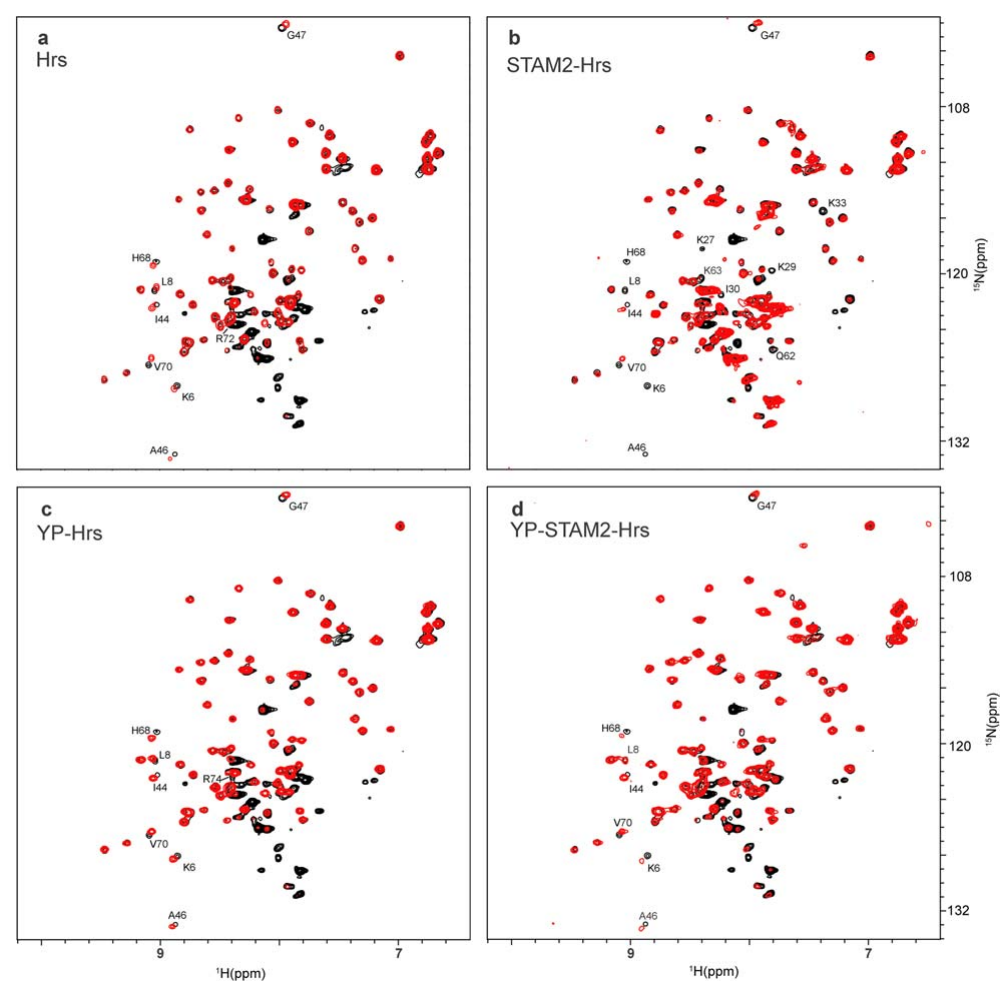
NMR-spectra of Ubiquitin-ligand complexes. ^1^H{^15^N}HSQC spectra of *E. coli* after 3-h of [^15^N]-Ubiquitin overexpression (black), overlaid with spectra (red) obtained from *E. coli* after 2-h of [^15^N]-Ubiquitin overexpression and: a) 4-h of Hrs overexpression; b) 4-h of STAM2 & Hrs co-overexpression; c) 4-h of Hrs and 2-h of Fyn kinase co-overexpression; d) 4-h of Hrs & STAM2 and 2-h of Fyn kinase co-overexpression. Individual peaks exhibiting either a chemical shift change >0.1 ppm or significant differential broadening (>30% change in intensity) are labeled with corresponding assignments. The strong peaks in the spectra between 8.5 and 7.8 ppm correspond to various metabolites of [U-^15^N] ammonium ion. NMR experiments were acquired at T = 298 K on Bruker Avance 700 MHz NMR spectrometer equipped with a cryoprobe. ^1^H{^15^N}-edited HSQC data were recorded with 16 transients as 512{128} complex points, apodized with a squared cosine-bell window function and zero-filled to 1k{256) points prior to Fourier transformation. The corresponding sweep widths were 12 and 35 ppm in the ^1^H and ^15^N dimensions, respectively. The Q49 peak is obscured by peaks from the [*U*-^15^N] ammonium ion metabolites and is not labeled. Ubiquitin ligands are indicated in each panel.

**Figure 3:**
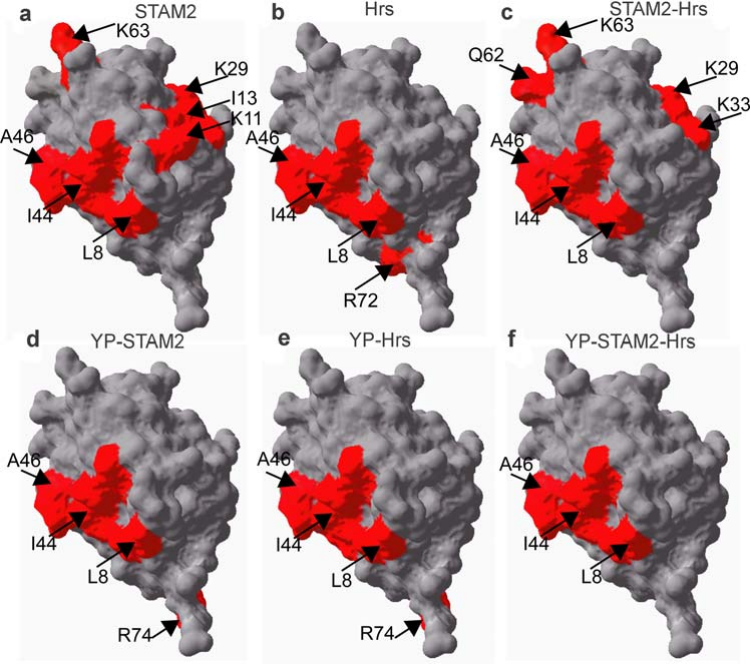
Interaction surface maps of Ubiquitin-ligand complexes. Interaction surface of Ubiquitin mapped onto the three-dimensional structure of Ubiquitin (PDB code 1D3Z). Individual residues exhibiting either a chemical shift change >0.05 ppm or significant differential broadening are indicated in red. All perturbed residues lie on the Ubiquitin surface and, therefore, reflect changes in the interaction surface of the molecule rather than changes in tertiary or quaternary structure. a) STAM2-Ubq interaction; b) Hrs-Ubq interaction; c) STAM2-Hrs-Ubq interaction; d) phosphorylated STAM2-Ubq interaction (YP-STAM2); e) phosphorylated Hrs-Ubq interaction (YP-Hrs); f) phosphorylated STAM2-Hrs-Ubq interaction (YP-STAM2-Hrs). Ubiquitin ligands are indicated in each panel.

To dissect changes in the interaction surface of Ubiquitin we created complexes of [*U*-, ^15^N]-Ubiquitin-STAM2, [*U*-, ^15^N]-Ubiquitin-Hrs, and [*U*-, ^15^N]-Ubiquitin-STAM2-Hrs (Fig. 2, Fig. S2). To study Ubiquitin binding to STAM2, we induced overexpression of [*U*-, ^15^N]-Ubiquitin prior to or following 3 or 4 hours of STAM2 overexpression. Over this time, the concentration of STAM2 was determined to range from 50–500 µM in these cells. As the concentration of STAM2 increased, we observed consistent broadening of selected Ubiquitin peaks in the NMR spectrum[5] (Fig. S2). The NMR solution structure of Ubiquitin, which consists of 76 amino acids, is well-known [15]. Mapping the differentially broadened peaks onto the three-dimensional structure of Ubiquitin results in two distinct surfaces that define the interface between Ubiquitin and STAM2[5] (Fig. 3a). The chemical shift changes of Ubiquitin that we observed affect only surface residues, implying that there are no conformational changes accompanying complex formation. The first interface corresponds to the UIM binding surface and consists of residues K6, L8, I44, A46, G47, H68 and V70; the second interface corresponds to the binding surface for the VHS domain of STAM2 and consists of K11, I13, K27, K29, K33 and K63. High affinity binding by STAM2 to Ubiquitin requires both the UIM and the VHS [14]. Since the overall binding affinity of Ubiquitin for STAM2 is ∼10 µM *versus* ∼100 µM for the UIM alone [5], we conclude that the second interacting surface is responsible for increasing the overall affinity of the interaction.

To study Ubiquitin binding to Hrs, we induced overexpression of [*U*-, ^15^N]-Ubiquitin prior to or following 3 or 4 hours of Hrs overexpression. Over this time, the concentration of Hrs was determined to range from 50–360 µM in these cells. As the concentration of Hrs increased, we observed changes in the chemical shifts of selected Ubiquitin peaks (Fig. 2a). Mapping the chemically-shifted peaks onto the three-dimensional structure of Ubiquitin results in a distinct interface between Hrs and Ubiquitin (Fig. 3b) corresponding to the UIM binding surface and consisting of residues K6, L8, I44, A46, G47, H68 and V70. In addition, R72, located near the C-terminus, was affected, resulting in an extension of the UIM binding patch. The reported binding affinity of Ubiquitin for Hrs is ∼200 µM; this is slightly weaker than the binding of an isolated UIM to Ubiquitin [18]. The UIM of Hrs is a variant of the conventional UIM helix, dubbed a double UIM (DUIM), and is capable of binding two Ubiquitin molecules [19]. The small changes in the NMR spectra observed for the interaction between the UIM of STAM2 and the DUIM of Hrs may result from an altered structure for the DUIM.

To study Ubiquitin binding to the STAM2-Hrs heterodimer, we induced overexpression of [*U*-, ^15^N]-Ubiquitin prior to or following 3 or 4 hours of STAM2 and Hrs co-overexpression. The concentrations of STAM2 and Hrs were determined to range from 50 to 280 µM and from 20 to 100 µM, respectively, in these cells. As the concentrations of STAM2 and Hrs increased, we observed consistent broadening of selected Ubiquitin peaks (Fig. 2b). Mapping the differentially broadened peaks onto the three-dimensional structure of Ubiquitin results in two distinct interfaces between Ubiquitin and STAM2-Hrs (Fig. 3c). The first interface corresponds to the UIM binding surface and includes residues K6, L8, I44, A46, G47, H68 and V70. The second interface includes residues K27, K29, K33, Q62 and K63, which are part of the Ubq-VHS contact surface, but not K11 and I13, which are perturbed in the interaction between Ubiquitin and STAM2. The change in the Ubq-VHS interaction surface is subtle, truncated at one end and slightly extended at the other, in a manner that is characteristic of the ternary complex and may be a result of STAM2 reorienting within the heterodimer relative to STAM2 alone since these residues are not affected when Hrs binds to Ubiquitin. Overall, the surface residues on Ubiquitin that interact with the UIM are virtually the same as those observed for the Ubq-STAM2 and Ubq-Hrs interactions, indicating that the binding of the UIM to Ubiquitin is largely unaltered.

### Binding phosphorylated interactor proteins results in the loss of contact surfaces on Ubiquitin

STAM2 and Hrs were phosphorylated by inducing overexpression of the constitutively active Src-family tyrosine-kinase, Fyn, for the final 2 hours of STAM2, Hrs, or STAM2-Hrs overexpression. The extent of STAM2 and Hrs phosphorylation were examined by using Western blots (Fig. 1d). Mass spectroscopic analysis (see Methods S1) of STAM2 revealed that Y291, Y371 and Y374 are phosphorylated by Fyn (Fig. S3). A major site of phosphorylation on STAM2 is reported to be Y192 [20], however, the absence of detectable phosphorylation at Y192 likely reflects the inability of Fyn to phosphorylate at that position. This is not unexpected since distinct non-receptor tyrosine kinases couple EGF, HGF and PDGF stimulation with tyrosine phosphorylation of STAM2 and Hrs *in vivo* [10]. Y371 and Y374 are located in the ITAM (immunoreceptor tyrosine-based activation motif) domain, which contains sites for phosphorylation by Src kinase. The ITAM domain has been identified as necessary for tyrosine phosphorylation of STAM2 by Jak1 [21].

We did not confirm phosphorylation sites in Hrs since these sites have been well-established. The major sites of EGF-dependent phosphorylation on human Hrs are Y329 and Y334 [22]. Tyrosine to phenylalanine mutations at these residues do not affect Ubq-Hrs binding, Hrs ubiquitination, or the activity of Hrs in the sorting mechanism [22]. Furthermore, an intact UIM is required for Hrs phosphorylation [22]. Extensive tyrosine phosphorylation of Hrs was not confirmed (Fig. 1d) suggesting that Fyn kinase is likely not the optimal kinase to phosphorylate Hrs. The extent of tyrosine phosphorylation on Hrs appeared to be enhanced by the presence of Ubiquitin (not shown). Indeed, it was previously noted that Hrs phosphorylation may require direct interaction between Ubiquitinated receptors and Hrs [10].

The STINT-NMR spectrum of Ubiquitin interacting with phosphorylated STAM2 reveals primarily changes in chemical shifts that affect a smaller number of residues than were perturbed in the Ubq-STAM2 interaction, and the loss of the interaction surface attributed to the VHS domain of STAM2 (Fig. S2c; Fig. 3d). That the NMR spectrum represents an interaction between Ubiquitin and phosphorylated STAM2 is implicit since a population of >70–80% modified interactor is required to generate a unique set of chemical shifts. We estimate that the affinity of Ubiquitin for phosphorylated STAM2 is >100 µM (refer to Materials and Methods), which is comparable to that of the isolated UIM. One additional residue, R74, was perturbed as a result of phosphorylated STAM2 binding to Ubiquitin suggesting a slight rearrangement of the UIM and possibly a change in the overall binding affinity.

To verify that the Ubiquitin residues perturbed when STAM2 is phosphorylated are due to the post-translational modification, we mutated the ITAM tyrosine residues, 371 and 374, to phenylalanines (Y371/4F-STAM2). When this mutant is overexpressed and its interaction with Ubiquitin examined by using STINT-NMR, the resulting spectra (Fig. S2) and surface interaction maps (Fig. S4) for both the unphosphorylated and phosphorylated states are largely identical to those obtained using unphosphorylated wild-type STAM2. Small differences between the spectra are likely due to the fact that at least one additional Fyn-dependent tyrosine phosphorylation site (Y291) was still present in the mutant. We conclude that phosphorylating the ITAM tyrosines weakens the binding between Ubiquitin and STAM2, and infer that these residues may be critical for the processivity of the internalizing pathway, attenuating the binding affinity of Ubiquitin for STAM2 in response to post-translational modification.

In spite of the fact that we did not confirm extensive phosphorylation of Hrs by Fyn kinase (Fig. 1d), the NMR spectrum of Ubiquitin interacting with Hrs changes when Fyn kinase is expressed in the same cells providing indirect evidence for Hrs phosphorylation. The STINT-NMR spectrum of Ubiquitin interacting with phosphorylated Hrs (Fig.2c) shows primarily changes in chemical shifts that affect a larger number of residues than were perturbed in the Ubq-Hrs interaction (Fig. 2a). When these changes are mapped to the Ubiquitin surface, K6, L8, I44, A46, G47, Q49, H68, V70 and R74, are seen to participate in the interaction, whereas R72 is no longer part of the interaction surface (Fig. 3e). The change in the chemical shift of R74, a residue that was also implicated in the interaction between Ubiquitin and phosphorylated STAM2, suggests that a similar alteration of the Ubq-UIM interaction occurs when Ubiquitin binds to either phosphorylated molecule. We estimate that the overall binding affinity of Ubiquitin for phosphorylated Hrs is >100 µM, which is comparable to the affinity of Ubiquitin for unphosphorylated Hrs and the isolated UIM. Thus, phosphorylating tyrosines on Hrs does not appreciably alter the binding of Ubiquitin to Hrs.

The STINT-NMR spectrum of Ubiquitin interacting with phosphorylated STAM2-Hrs reveals changes in chemical shifts for a number of residues (Fig. 2d) and the loss of the interacting surface attributed to the VHS domain of STAM2 (Fig. 3f). The residues associated with UIM binding to Ubiquitin (K6, L8, I44, A46, G47, H68 and V70) are perturbed. However, residue R74, which is chemically shifted in the interaction between Ubiquitin and phosphorylated STAM2 or phosphorylated Hrs alone, is not perturbed. Ubiquitin appears to interact with the phosphorylated ternary complex in much the same way that it interacts with phosphorylated STAM2 and phosphorylated Hrs, involving contact with only the UIMs of both interactor proteins and a commensurate weakening of the binding due to the loss of the second interaction surface.

## Discussion

There are advantages to using in-cell rather than *in vitro* NMR experiments to study interacting proteins: 1) the proteolytic machinery in cells is tightly regulated and this regulation is lost in lysates, which can result in proteolysis of the sample; 2) in-cell protein overexpression results in higher local concentrations of interacting partners than in lysates, thus increasing the likelihood of detecting weak interactions; 3) interactions occur within a cellular environment, which may confer biologically relevant structural conformations that cannot be duplicated *in vitro*.

Hrs and, to a lesser extent, STAM2, are somewhat labile. Therefore working with purified protein and performing *in vitro* binding assays are not feasible for either of these species. Furthermore, *in vitro* binding assays will not provide any structural details about the interaction, but will merely provide a more precise estimate of binding affinities. While the affinities that we estimated are consistent with those reported from *in vitro* studies(Shekhtman, 2002), we are primarily concerned with the interaction surfaces on Ubiquitin and how modulating these surfaces affects the processivity of the internalizing pathway.

Applying *in-cell* biochemistry using NMR spectroscopy to the ternary Ubiquitin-Hrs-STAM2 complex showed that the interaction surface is significantly modulated by the phosphorylation state of two STAM2 tyrosines, Y371 and Y374, located in the conserved ITAM domain. Since the ITAM domain is in immediate proximity to the STAM2 homo- and hetero-dimerization domain GAT [23], we expect that a change in the phosphorylation state of Y371 and Y374 will cause intermolecular rearrangements of the Ubiquitin-binding domains, VHS and UIM, leading to the observed loss of the VHS binding surface.

Our data cannot distinguish whether Ubiquitin binds separately to the VHS and to the UIM (two Ubiquitins bound per STAM2) or if the two binding motifs interact cooperatively to increase the overall affinity of Ubiquitin over that of the binding to either motif separately. During endocytosis, the bound Ubiquitin may come from the free cellular pool or from monoubiquitinated substrates. The latter form of the ligand may serve to “cross link” the higher order species formed, and may bind with a different affinity from free ubiquitin.

The study of biochemistry inside living cells entails utilizing complementary methods to resolve processes on different scales from micrometers to Angstroms. Light and fluorescence microscopy were successfully applied to study the localization and compartmentalization of macromolecules *in vivo* on the micrometer scale. Förster resonance energy transfer (FRET) extended our ability to identify protein-protein and protein-nucleic acid interactions on a scale of tens of nanometers and facilitated estimates of binding affinities and stoichiometries [24]. The advent of in-cell biochemistry using STINT-NMR, which allows us to modify and examine protein-protein interaction surfaces at the level of single amino acid residues, has pushed the limits of resolution to the subnanometer scale. Though in its infancy, this technique has the potential to open a window for investigating life processes inside a living cell at a level of detail never seen before.

## Materials and Methods

### Sequential over-expression and labeling

*E.coli* strain BL21(DE3) codon+[Novagen] was co-transformed with pASK-Ubq and: pCDF-ST2 (Ubq-STAM2 interaction); or pRSF-Hrs (Ubq-Hrs interaction); or pCDFDuet-Hrs-ST2 (Ubq-STAM2-Hrs interaction); or pCDF-ST2 and pDB1-Fyn (Ubq-phosphorylated STAM2 interaction); or pCDFDuet-Hrs and pDB1-Fyn (Ubq-phosphorylated Hrs interaction); or pCDFDuet-Hrs-ST2 and pDB1-Fyn (Ubq-phosphorylated STAM2-Hrs interaction). (For details of plasmid constructions see Methods S1).

Cells were grown overnight at 37°C to an OD_600_ of ≥1.6 in Luria-Bertani medium (LB) supplemented with 150 mg/L of carbenicillin for cultures containing pASK-Ubq, 35 mg/L of kanamycin for cultures containing pRSF-Hrs or pDB1-Fyn, and 50 mg/L of streptomycin for cultures containing pCDF-ST2 or pCDFDuet-Hrs or pCDFDuet-Hrs-ST2. Cultures containing pDB1-Fyn were supplemented with 0.2% glucose to suppress *fyn* transcription from the P_BAD_ promoter.

Two protocols were employed: In the first, the Ubiquitin target was overexpressed and labeled followed by overexpression of the interactor(s) and Fyn kinase; in the second, the interactor(s) and Fyn kinase were overexpressed followed by overexpression and labeling of the Ubiquitin target. The first protocol yielded a high concentration ratio of labeled target to interactor, while the second yielded a low ratio of target to interactor. Both protocols were required to assess the endpoint of the structural transition.

### Protocol 1: Expression of [U-*^15^N*]-Ubiquitin

Cells from the overnight culture were washed once with minimal medium (M9) salts and re-suspended to an OD_600_ of ∼0.5 in M9 medium containing the appropriate antibiotics, [*U*-^15^N] ammonium chloride (0.7 g/L) as the sole nitrogen source and either 0.2% glucose (for cultures containing pDB1-Fyn) or 0.4% glycerol as the sole carbon source. *N.b.* For all induced cultures we substituted ampicillin (100 mg/L) for carbenicillin. The cells were incubated at 37°C for 10–15 minutes and Ubiquitin overexpression was induced by adding 2 mg/mL anhydrotetracycline in dimethylformamide to a final concentration of 0.2 µg/mL. Ubiquitin overexpression was allowed to proceed for up to 4 hours.

Following the first induction, a 100 mL sample of culture was collected, the cells were centrifuged, washed twice with 50 mL of 10 mM potassium phosphate buffer [pH 7], resuspended with 1 mL 10 mM potassium phosphate buffer [pH 7.0] containing 10% glycerol and stored at −80°C for subsequent NMR analysis. This control sample was used to assess the extent of overexpression and quality of labeling for a given experiment.

### Expression of STAM2, Hrs and Fyn kinase domain

Following Ubiquitin overexpression and labeling, the culture was centrifuged and washed once with M9 salts before resuspending a sufficient number of cells to yield an OD_600_ of ∼0.5 in LB medium supplemented with the appropriate antibiotics. The culture was incubated at 37°C for 10–15 minutes and 0.5 M IPTG was added to a final concentration of 0.5 mM to induce individual over-expression of STAM2 or Hrs, or 2 mM to induce simultaneous overexpression of STAM2 and Hrs; induction was allowed to proceed for 3 or 4 hours. In phosphorylation experiments, following 1 or 2 hours of IPTG induction, 20% L-arabinose was added to a final concentration of 0.2%, and overexpression of the Fyn kinase was allowed to proceed for 2 hours. 100 mL samples were taken, centrifuged, washed twice with 10 mM potassium phosphate buffer [pH 7], resuspended with 1 mL 10 mM potassium phosphate buffer [pH 7.0] containing 10% glycerol and stored at −80°C for subsequent NMR analysis. The use of a cryoprotectant is critical to eliminate cell lysis or breakage due to repeated freeze-thawing.

### Protocol 2: Expression of STAM2, Hrs and Fyn kinase domain

Cells from the overnight culture were washed once with minimal medium (M9) salts and re-suspended to an OD_600_ of ∼0.5 in LB medium supplemented with the appropriate antibiotics. The culture was incubated at 37°C for 10–15 minutes and 0.5 M IPTG was added to a final concentration of 0.5 mM to induce individual over-expression of STAM2 or Hrs, or 2 mM to induce simultaneous overexpression of STAM2 and Hrs; induction was allowed to proceed for 3 or 4 hours. In phosphorylation experiments, following 1 or 2 hours of IPTG induction, 20% L-arabinose was added to a final concentration of 0.2%, and overexpression of the Fyn kinase was allowed to proceed for 2 hours.

Separately, a sufficient volume of overnight culture was centrifuged, washed once with minimal medium (M9) salts and re-suspended to an OD_600_ of ∼0.5 in M9 medium containing the appropriate antibiotics, [*U*-^15^N] ammonium chloride (0.7 g/L) as the sole nitrogen source and either 0.2% glucose (for cultures containing pDB1-Fyn) or 0.4% glycerol or as the sole carbon source. The cells were incubated at 37°C for 10–15 minutes and Ubiquitin over-expression was induced by adding 2 mg/mL anhydrotetracycline in dimethylformamide to a final concentration of 0.2 µg/mL. After 4 hours of induction, a 100 mL sample of culture was collected, centrifuged, washed twice with 50 mL of 10 mM potassium phosphate buffer [pH 7], resuspended with 1 mL 10 mM potassium phosphate buffer [pH 7.0] containing 10% glycerol and stored at −80°C for subsequent NMR analysis. This control sample was used to assess the extent of overexpression and quality of labeling for a given experiment.

### Expression of [U-*^15^N*]-Ubiquitin

Following overexpression of the interactor (with or without post-translational modification), the culture was centrifuged and washed once with M9 salts before resuspending a sufficient number of cells to yield an OD_600_ of ∼0.5 in M9 medium containing the appropriate antibiotics, [*U*-^15^N] ammonium chloride (0.7 g/L) as the sole nitrogen source and either 0.2% glucose (for cultures containing pDB1-Fyn.) or 0.4% glycerol as the sole carbon source. The cells were incubated at 37°C for 10–15 minutes and Ubiquitin over-expression was induced by adding 2 mg/mL anhydrotetracycline in dimethylformamide to a final concentration of 0.2 µg/mL. Ubiquitin over-expression was allowed to proceed for 2, 3 or 4 hours. 100 mL samples were taken, centrifuged, washed twice with 10 mM potassium phosphate buffer [pH 7], resuspended with 1 mL 10 mM potassium phosphate buffer [pH 7.0] containing 10% glycerol and stored at −80°C for subsequent NMR analysis. The use of a cryoprotectant is critical to eliminate cell lysis or breakage due to repeated freeze-thawing.

### Quantitation of intracellular STAM2 and Hrs

To estimate the concentration of overexpressed STAM2 and Hrs present in cells, 10% SDS-PAGE was performed. Each gel contained a concentration range of purified STAM2 to generate a standard curve and samples from induced cells for each of the overexpression time points used in the STINT-NMR titration experiments. Gels were stained with SYPRO Ruby Gel Stain (Bio-Rad), which provides a linear fluorescent response over a wide range of protein concentration, and scanned using a Typhoon Trio Variable Mode Imager (Amersham). The scanned gels were analyzed using ImageQuant5.2 software (Molecular Dynamics). Blocked bands were corrected for background and the unknown samples were also corrected by subtracting the fluorescent optical density of a sample that was not induced for protein overexpression. We assumed 4.4×10^8^ cells per mL per optical density unit at 600 nm and a cell volume of 4.2×10^−15^ L, modeled as a sphere of 2 µm in diameter. The experiments were performed in duplicate.

### NMR spectroscopy

[*U*-^15^N] labeled cells were re-suspended in 0.5 mL of NMR buffer (10 mM potassium phosphate, pH 7.0, 90%/10% H_2_O/D_2_O) and transferred to an NMR tube. To rule out the possibility that the visible NMR spectrum was due to extracellular proteins due to leakage from the cells, we sedimented the cells from the NMR sample and acquired the ^1^H{^15^N}-HSQC spectrum of the resultant supernatant (Figure S1). No protein NMR signal was visible above the noise level. All NMR experiments were performed using a Bruker Avance 700 MHz NMR spectrometer equipped with a cryoprobe. The cryoprobe affords a four-fold increase in sensitivity allowing data collection within ∼1 hr for an individual experiment; this is critical to minimize cell leakage [17]. We used a watergate version of the ^1^H{^15^N}-HSQC spectrum. ^1^H{^15^N}-edited HSQC data were recorded with 32 transients as 512{64} complex points, apodized with a squared cosine-bell window function and zero-filled to 1k{128} points prior to Fourier transformation. The corresponding sweep widths were 12 and 35 ppm in the ^1^H and ^15^N dimensions, respectively. Chemical shifts of [*U*-, ^15^N]-Ubiquitin inside the cell are slightly different from purified Ubiquitin. We reassigned the backbone chemical shifts of Ubiquitin using the clarified lysate of [U-, ^13^C, ^15^N]-Ubiquitin and a standard suite of triple resonance experiments [25]. During in-cell titration experiments, we measured the change in the chemical shifts of amide nitrogens and covalently attached amide protons according to the equation: 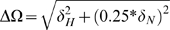, where *δ_H_*_(*N*)_ represents a change in hydrogen and nitrogen chemical shifts. Even without quantifying protein concentrations present in the cell, in-cell NMR spectroscopy allows us to make a crude estimate of the protein binding affinities. Depending on the magnitude of the chemical shift change ΔΩ and the rate constant, *k_off_*, between bound and free states, chemical exchange can result in gradual changes of chemical shifts when ΔΩ<<*k_off_* (fast exchange), line broadening when ΔΩ≤*k_off_* (intermediate exchange) or the appearance of new peaks when ΔΩ>>*k_off_* (slow exchange). Assuming that the binding reaction is diffusion limited and the average change of the chemical shift is ∼0.01 ppm, the fast exchange regime will occur when the dissociation constant, K_d_, is larger than 100 µM and intermediate or slow exchange will occur when the dissociation constant is less than or equal to 10 µM.

## Supporting Information

Methods S1 Supplementary Methods
(0.03 MB DOC)

Figure S1 (0.32 MB DOC)

Figure S2 (1.38 MB DOC)

Figure S3 (0.83 MB DOC)

Figure S4 (1.27 MB DOC)
