# Supplementary material for: In-Cell Biochemistry Using NMR Spectroscopy
Source: PLoS One. 2008 Jul 2;3(7):e2571. doi: 10.1371/journal.pone.0002571 (PMC2453524; doi:10.1371/journal.pone.0002571)
Supplement: Methods S1 — Supplementary Methods (0.03 MB DOC) [file pone.0002571.s001.doc]

**METHODS S1**

***Plasmid constructs.***

***pASK-Ubq***. DNA coding for full-length Ubiquitin (amino acids 1-76) was amplified from pBAD-Ubq [Burz *et al*. Nat Methods 3, 91 (Feb, 2006)] using the oligonucleotides 5'-CCCCCCGGTCTCTAATGGCAATCTTCGTCAAGACGTTAACCGG-3' and 5'-CCCCCCGGTCTCTGCGCTTCAACCACCTCTTAGTCTTAAGACAAGATGTAAGG-3'. The gene was ligated into pASK-IBA3+ [IBA] using the *Bsa*I linker sites. The resulting plasmid, pASK-Ubq, expresses Ubiquitin from a tet promoter/operator, which is induced by tetracycline or anhydrotetracycline. This plasmid confers ampicillin resistance and contains an f1 origin and the *tet* gene, which codes for Tet repressor.

***pRSF-Hrs***. DNA coding for full-length (human) Hrs was amplified from pMAL-Hrs (from Dr. H. Stenmark, Institute for Cancer Research, Norway) using the oligonucleotides 5'-CATCGTGTCGACTCAGTCAAAGGAGATGAGCTGGG-3' and 5'-TTGGATCCCATGGGGCGAGGCAGCGGCAC-3'. The gene was ligated into pRSF-1b [Novagen] using the *BamH*I and *Sal*I linker sites. The resulting plasmid, pRSF-Hrs, expresses Hrs from a T7 promoter/lac operator (P_T7_/lacOp), which is induced by isopropyl-β-D-thiogalactoside (IPTG). This plasmid confers kanamycin resistance and contains an RSF replication origin and the *lacI* gene, which codes for Lac repressor.

***pCDFDuetHrs-ST2***. DNA coding for full-length Hrs was restriction digested from pRSF-Hrs using *BamH*I and *Sal*I and ligated into pCDFDuet-1 [Novagen]. The resulting plasmid, pCDFDuet-Hrs, expresses Hrs from P_T7_/lacOp and is induced by IPTG. DNA coding for full-length murine STAM2 was amplified from pGEX-STAM2 (from Dr. H. Stenmark, Institute for Cancer Research, Norway) using the oligonucleotides 5'-GGAATTCCATATGCCTCTGTTCACTGCCAACCC-3' and 5'-CCCTTTTTAATTAACTACAGGAGAGGCTGCTGGTG-3'. The gene was ligated into pCDFDuet-Hrs using the *Nde*I and *Pac*I linker sites. The resulting plasmid, pCDFDuet-Hrs-ST2, expresses Hrs and STAM2 from separate P_T7_/lacOp's, both of which are induced by IPTG. This plasmid confers streptomycin resistance and contains a CDF replication origin and the *lacI* gene, which codes for Lac repressor.

***pBAD-Fyn***. DNA coding for human Fyn kinase domain (amino acids 271-537) was amplified from pGEX-Fyn (from Dr. K.M. Shokat, UC San Francisco) using the oligonucleotides 5'-TTTTTTCCATGGCACAAACTTCTGGATTGGCTAAAGATGCTTGG-3' and 5'-CCCAAGCTTTCACAGGTTTTCACCGGGCTGATACTGG-3'. The 800 nt PCR product was ligated into pBAD202 [Invitrogen] using the *Nco*I and *Hind*III linker sites. The resulting plasmid, pBAD-Fyn, expresses the Fyn kinase domain from the *araBAD* promoter/operator (P_BAD_), which is induced by L-arabinose. This plasmid confers ampicillin resistance and contains a pUC replication origin and the *araC* gene, which codes for AraC repressor.

***pDB1***. pBAD202 was restriction digested with *Nde*I and pRSF-1b was restriction digested with *Xba*I; both were blunt-ended by filling in using the Klenow fragment [Bio-Labs] and then both were restriction digested using *Hind*III. A 1998 bp fragment was isolated from the pBAD202 digest and a 2075 bp fragment from the pRSF digest. Ligation of the two fragments yielded pDB1, a 4073 bp construct that confers kanamycin resistance and contains the *ara*C gene, P_BAD_, the pBAD202 linker region through the *Hind*II site and an RSF origin.

***pDB1-Fyn***. DNA coding for human Fyn kinase domain (amino acids 271-537) was amplified from the 800 nt PCR product used to clone the *fyn* kinase domain into pBAD202 using the same oligonucleotides used to clone Fyn into pBAD. The gene was ligated into pDB1 using the *Nco*I and *Hind*III linker sites. The resulting plasmid, pDB1-Fyn, expresses the Fyn kinase domain from P_BAD_ and is induced by L-arabinose. This plasmid confers kanamycin resistance, contains an RSF replication origin and the *araC* gene, which codes for AraC repressor.

***Mass spectrometry.*** Phosphorylated protein was electrophoresed and the bands cut out for MS analysis. The gel pieces were washed, reduced, alkylated and in-gel tryptic digested. Proteolytic peptides were extracted from the gel followed by TiO_2_ IMAC enrichment for the phosphopeptide. The flow through and elution fractions were concentrated and reconstituted in 5% formic acid for LC-MS/MS analysis. Chromatography was performed using a CapLC HPLC column (Waters) and a Magic C18 column (Michrom Bioresources). Elution was accomplished using a step linear gradient from 3% acetonitrile, 0.1% formic acid and 0.01% trifluoroacetic acid (TFA) to 100% acetonitrile, 0.1% formic acid and 0.01% TFA. MS/MS was performed using a Q-TOF2 instrument (Waters/Micromass). PKL files were created for the flow-through and eluted samples using Masslynx 3.5 software (Waters). MASCOT 2.1 (Matrix Science) software was used to correlate the tandem mass spectra data to the protein sequence.
