# Supplementary material for: In-Cell Biochemistry Using NMR Spectroscopy
Source: PLoS One. 2008 Jul 2;3(7):e2571. doi: 10.1371/journal.pone.0002571 (PMC2453524; doi:10.1371/journal.pone.0002571)
Supplement: Figure S1 — (0.32 MB DOC) [file pone.0002571.s002.doc]

**
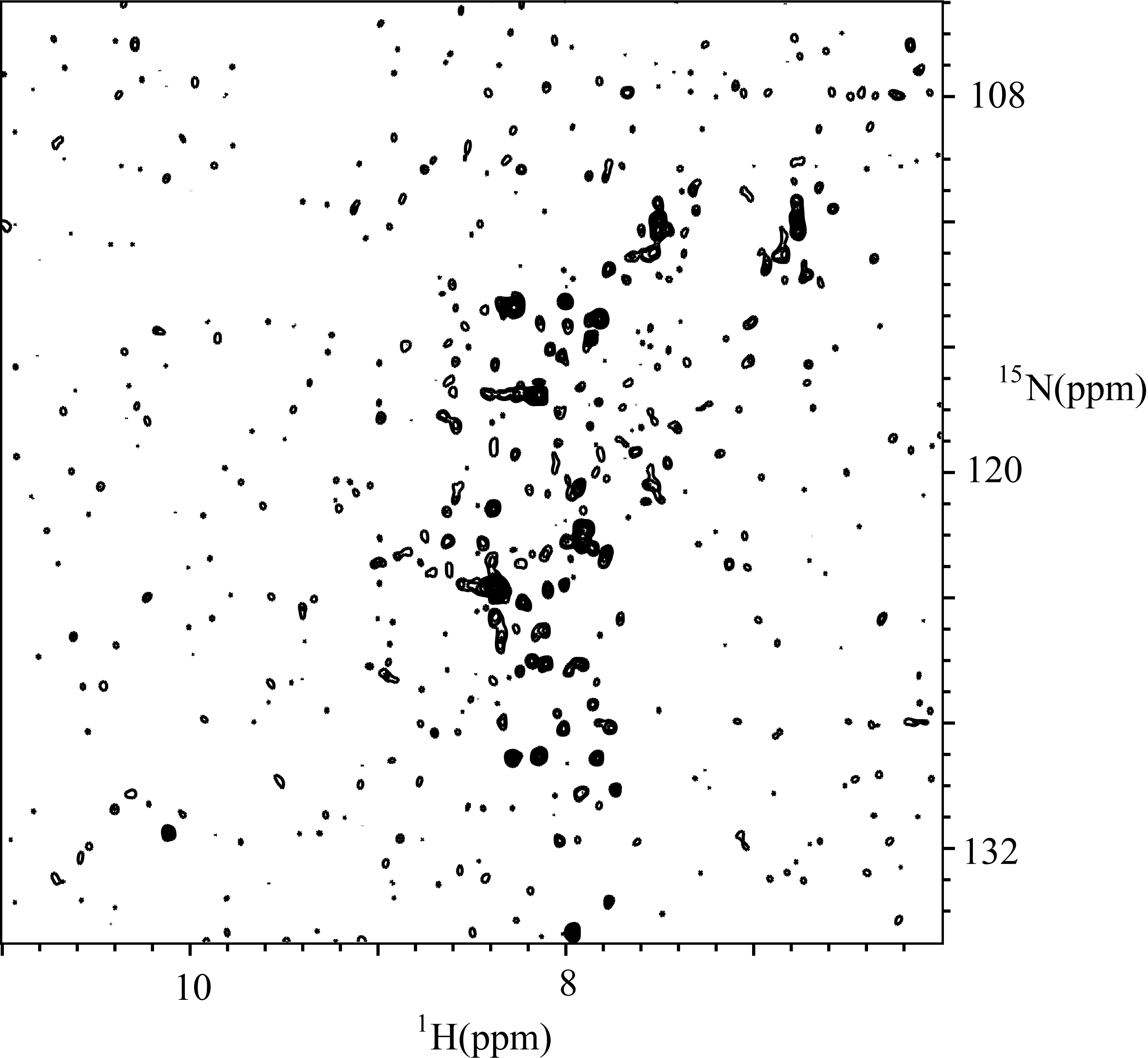
**

**Figure S1. NMR spectrum of cell suspension supernatant.** Negative control showing that the in-cell NMR spectra are due to intracellular Ubiquitin. After obtaining the in-cell NMR spectrum, the cells were centrifuged and the supernatant was examined. No NMR spectrum of Ubiquitin was observed above noise level, implying that there is no leakage or cell lysis occurring during the time it takes to acquire the NMR spectrum. The strong peaks in the spectrum between 8.5 and 7.8 ppm correspond to various metabolites of [U-^15^N] ammonium ion. NMR experiments were acquired at T=298 K on Bruker Avance 700 MHz NMR spectrometer equipped with a cryoprobe. ^1^H{^15^N}-edited HSQC data were recorded with 16 transients as 512{128} complex points, apodized with a squared cosine-bell window function and zero-filled to 1k{512) points prior to Fourier transformation. The corresponding sweep widths were 12 and 35 ppm in the ^1^H and ^15^N dimensions, respectively.
