# Supplementary material for: In-Cell Biochemistry Using NMR Spectroscopy
Source: PLoS One. 2008 Jul 2;3(7):e2571. doi: 10.1371/journal.pone.0002571 (PMC2453524; doi:10.1371/journal.pone.0002571)
Supplement: Figure S2 — (1.38 MB DOC) [file pone.0002571.s003.doc]

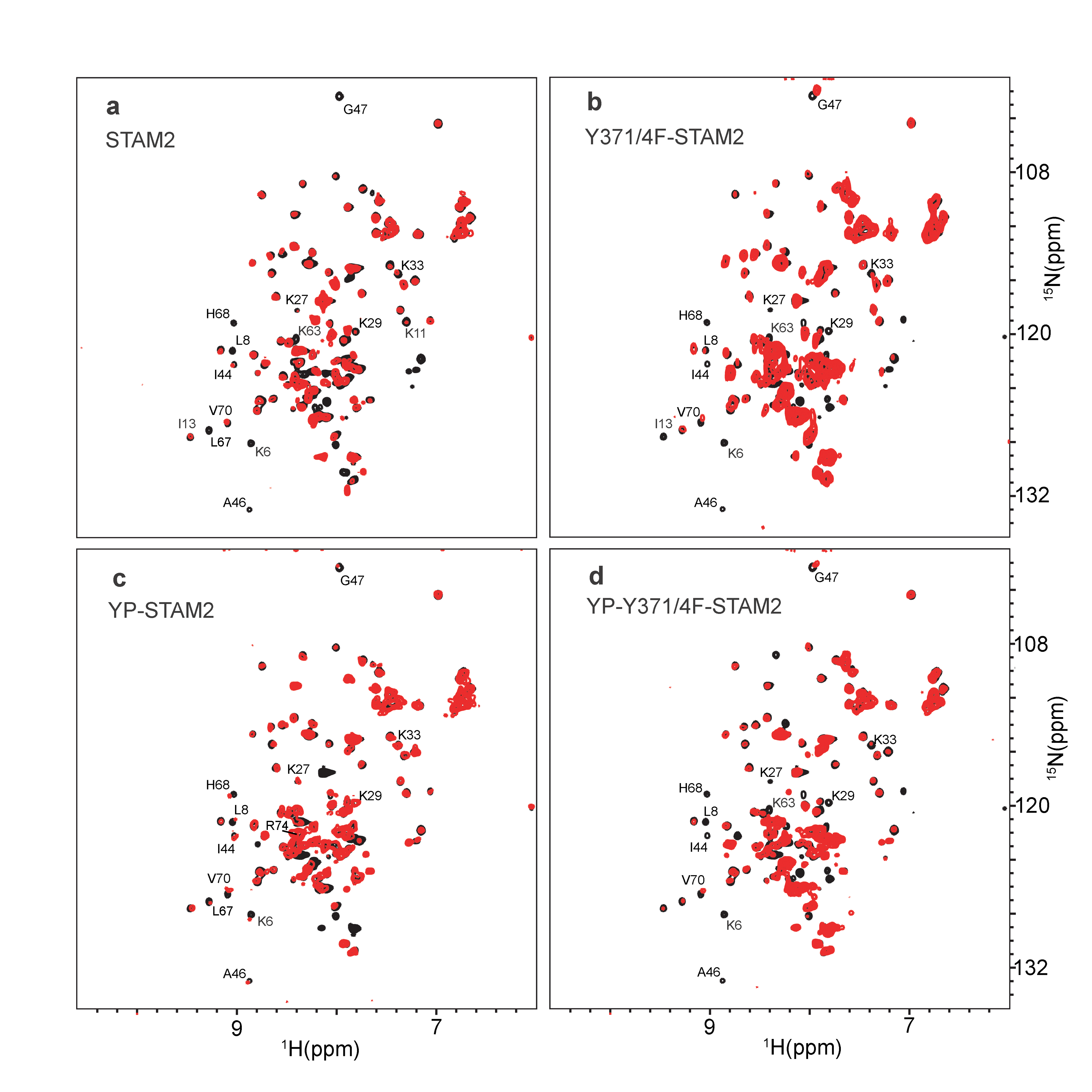


**Figure S2.** **NMR-spectra of Ubiquitin-ligand complexes.** ^1^H{^15^N}HSQC spectra of *E. coli* after 3-h of [^15^N]-Ubiquitin overexpression (black), overlaid with spectra (red) obtained from *E. coli* after 2-h of [^15^N]-Ubiquitin overexpression and: a) 4-h of STAM2 overexpression; b) 4-h of Y371/4F-STAM2 overexpression; c) 4-h of STAM2 and 2-h of Fyn kinase co-overexpression (YP-STAM2); d) 4-h of Y371/4F-STAM2 and 2-h of Fyn kinase co-overexpression (YP-Y371/4F-STAM2). Individual peaks exhibiting either a chemical shift change >0.1 ppm or significant differential broadening (>30% change in intensity) are labeled with corresponding assignments. The strong peaks in the spectra between 8.5 and 7.8 ppm correspond to various metabolites of [U-^15^N] ammonium ion. NMR experiments were acquired at T=298 K on Bruker Avance 700 MHz NMR spectrometer equipped with a cryoprobe. ^1^H{^15^N}-edited HSQC data were recorded with 16 transients as 512{128} complex points, apodized with a squared cosine-bell window function and zero-filled to 1k{512) points prior to Fourier transformation. The corresponding sweep widths were 12 and 35 ppm in the ^1^H and ^15^N dimensions, respectively. Ubiquitin ligands are indicated in each panel.
