# Supplementary material for: In-Cell Biochemistry Using NMR Spectroscopy
Source: PLoS One. 2008 Jul 2;3(7):e2571. doi: 10.1371/journal.pone.0002571 (PMC2453524; doi:10.1371/journal.pone.0002571)
Supplement: Figure S3 — (0.83 MB DOC) [file pone.0002571.s004.doc]

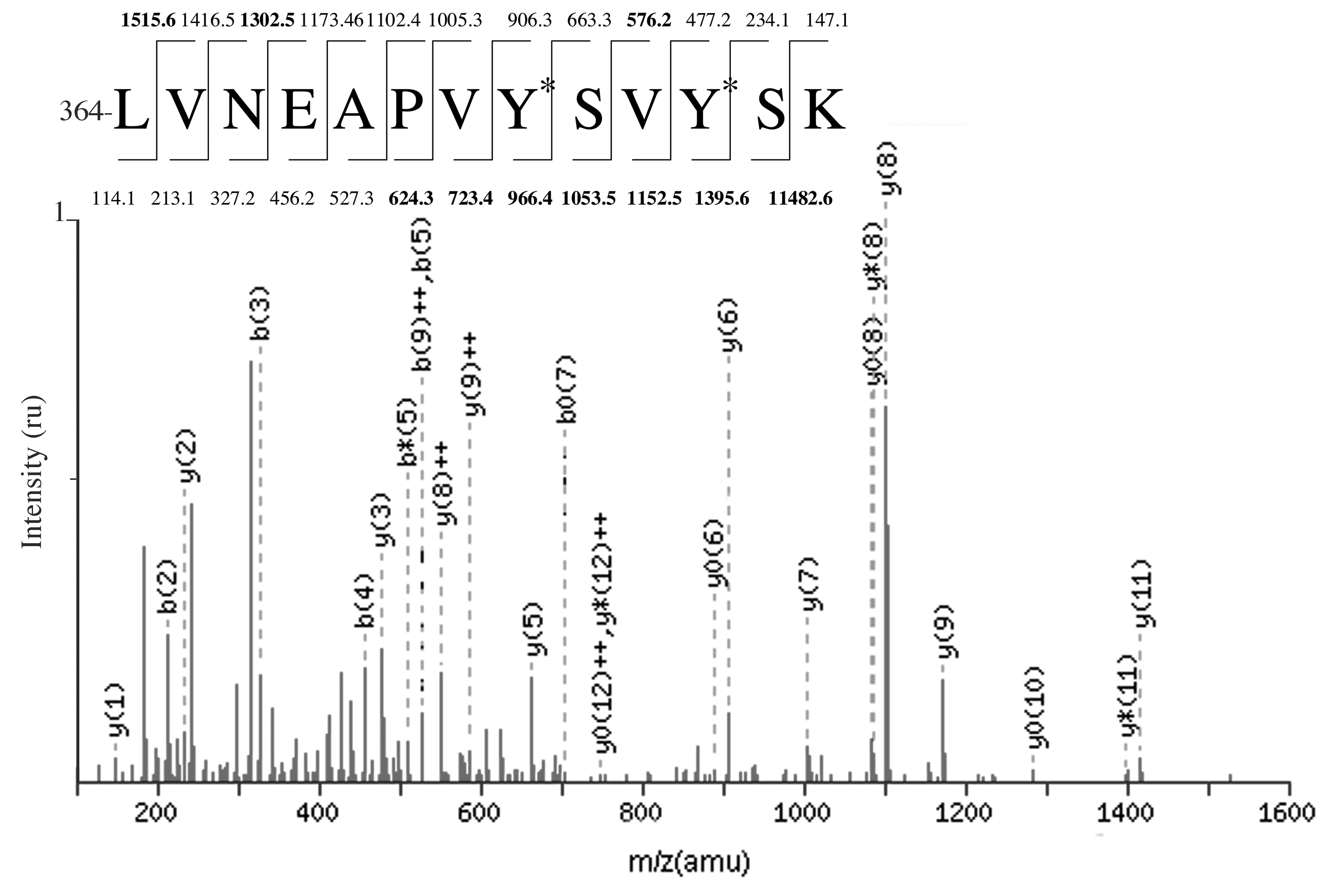


**Figure S3.** **MS/MS spectrum and sequence of tyrosine phosphorylated peptide from the ITAM motif of STAM2.** The *y** or *b** ions represent *y* or *b* fragment ions caused by loss of ammonia. The *y0* or *b0* ions represent *y* or *b* fragment ions caused by loss of H_2_O. The mass values for *y* and *b* fragment ions are shown above and below the peptide sequence, respectively. The mass values of doubly charged fragment ions in the peptide sequence are shown in bold.
