# Supplementary material for: In-Cell Biochemistry Using NMR Spectroscopy
Source: PLoS One. 2008 Jul 2;3(7):e2571. doi: 10.1371/journal.pone.0002571 (PMC2453524; doi:10.1371/journal.pone.0002571)
Supplement: Figure S4 — (1.27 MB DOC) [file pone.0002571.s005.doc]

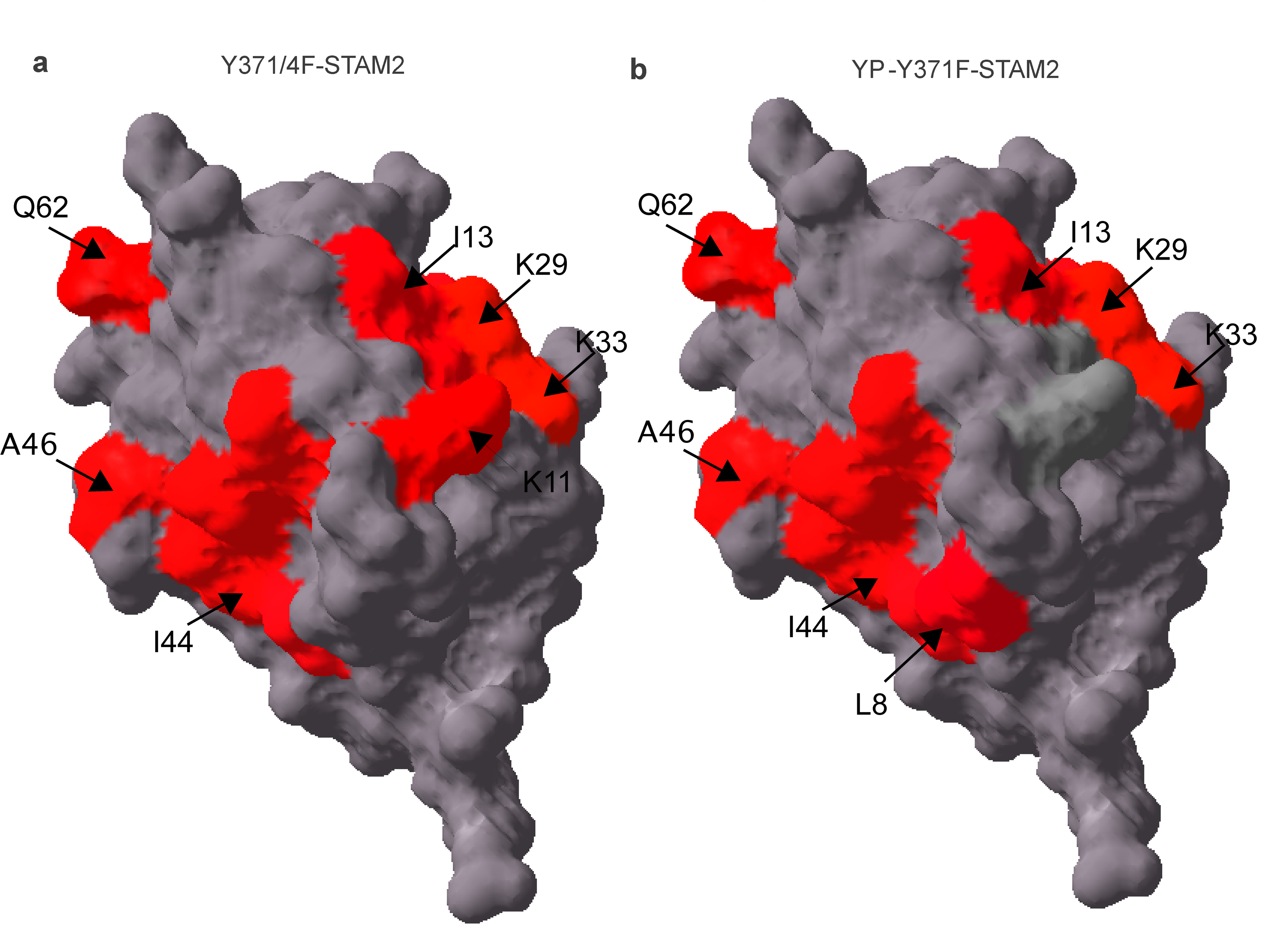


**Figure S4. Interaction surface maps of Ubiquitin-ligand complexes.** Interaction surface of Ubiquitin mapped onto the three-dimensional structure of Ubiquitin (PDB code 1D3Z). Individual residues exhibiting either a chemical shift change >0.05 ppm or significant differential broadening are indicated in red. All perturbed residues lie on the Ubiquitin surface and, therefore, reflect changes in the interaction surface of the molecule rather than changes in tertiary or quaternary structure. a) Y371/4F-STAM2-Ubq interaction; b) phosphorylated Y371/4F-STAM2-Ubq interaction (YP-Y371/4F-STAM2). Ubiquitin ligands are indicated in each panel.
